# Incidence of Idiopathic Intracranial Hypertension in Individuals With Gonadotropin-Releasing Hormone Analogue Treatment for Gender Dysphoria in Sweden

**DOI:** 10.1001/jamapediatrics.2023.0656

**Published:** 2023-05-01

**Authors:** Georgios Karamanis, Thomas Frisell, Mats Holmberg, Maria Halldin, Sara Sylvén, Alkistis Skalkidou, Fotios C. Papadopoulos

**Affiliations:** 1 Department of Medical Sciences, Psychiatry, Uppsala University Hospital, Uppsala, Sweden; 2 Clinical Epidemiology Division, Department of Medicine Solna, Karolinska Institutet, Stockholm, Sweden; 3 ANOVA, Karolinska University Hospital, Stockholm, Sweden; 4 Department of Women's and Children's Health, Karolinska Institutet, Stockholm, Sweden; 5 Department of Women's and Children's Health, Obstetrics and Gynecology, Uppsala University, Uppsala, Sweden

## Abstract

This cohort study examines the incidence of idiopathic intracranial hypertension (IHH) among individuals in Sweden undergoing gonadotropin-releasing hormone analogue (GnRHa) treatment for gender dysphoria.

In 2022, the US Food and Drug Administration (FDA) updated the safety labels for gonadotropin-releasing hormone analogues (GnRHa) with a warning about the risk of idiopathic intracranial hypertension (IIH),^1^ a rare condition in which elevated cerebrospinal fluid pressure can lead to symptoms such as severe headache and vision loss.^2^ The warning was added after 6 patients developed IIH after GnRHa treatment.^1^ In this study, we assessed the incidence of IIH in individuals diagnosed with gender dysphoria (GD) in Sweden, with and without GnRHa treatment.

## Methods

Using data from Swedish national registers (eAppendix in Supplement 1), we identified individuals with at least 1 GD diagnosis between 2006 and 2016. For every included individual, we selected 20 individuals without a GD diagnosis and matched for age, same- and opposite-sex assignment at birth, and county of residence. Follow-up started at first GD diagnosis or GnRHa initiation and was censored at death or emigration from Sweden. Details on the subcohorts are shown in Table 1. Incidence rates of IIH (first *International Statistical Classification of Diseases and Related Health Problems, Tenth Revision* code G93.2 set during inpatient or specialist outpatient care) with 95% CIs were calculated assuming a Poisson distribution. This cohort study was approved by the Central Ethical Review Board in Stockholm and informed consent was waived because the data the study was register-based and data on individuals were deidentified. We followed the STROBE reporting guideline and analyzed data from July 20, 2022, to February 17, 2023, using R software, versions 4.2.1 and 4.2.2 (R Foundation for Statistical Computing).

**Table 1.** Description of the Subcohorts, Inclusion and Exclusion Criteria, and Entry and End Dates of Follow-up Used to Calculate Person-Time of Exposure

Cohort	Inclusion criteria	Exclusion criteria	Entry date	End date of follow-up
GD with GnRHa	At least 1 GD diagnosis (*ICD-10* codes F64.0, F64.8, or F64.9) in the NPR and treatment with GnRHa (ATC codes H01CA or L02AE in the PDR) after or within 2 y before the first GD diagnosis	IIH diagnosis (*ICD-10* code G93.2 in the NPR) before GD diagnosis	Date of first dispensed prescription for GnRHa	The earliest date of first IIH diagnosis, date of death, date of emigration, or end of study^a^
GD without GnRHa	At least 1 GD diagnosis	IIH diagnosis before GD diagnosis	Date of first GD diagnosis	The earliest date of first IIH diagnosis, first dispensed GnRHa prescription, date of death, date of emigration, or end of study^a^
General population without GD or GnRHa	20 Randomly selected individuals from general population for each selected individual with GD, matched for age, same- and opposite- assigned sex at birth, and county of residence	Lifetime GD diagnosis, IIH diagnosis before entry date, or GnRHa prescription before entry date	Date of first GD diagnosis of the matched person	The earliest date of first IIH diagnosis, first dispensed GnRHa prescription, date of death, date of emigration, or end of study^a^

Abbreviations: ATC, Anatomical Therapeutic Chemical; GD, gender dysphoria; GnRHa, gonadotropin-releasing hormone analogue; *ICD-10, International Statistical Classification of Diseases and Related Health Problems, Tenth Revision;* IIH, idiopathic intracranial hypertension; NPR, National Patient Register; PDR, Prescribed Drug Register.

^a^ End of study was December 31, 2016.

## Results

The 410 individuals in the GD cohort who received GnRHa (211 [51%] assigned female at birth and 199 [49%] assigned male at birth) were younger (median [range] age, 17.8 [10.7-75.6] years) than 3820 individuals with GD without GnRHa (2085 [55%] assigned female at birth and 1735 [45%] assigned male at birth; median [range] age, 23.1 [8.0-94.2] years) (Table 2). The general population without GD was well matched.

**Table 2.** Descriptive Statistics and Incidence Rates for IIH in the 3 Subcohorts Studied

	Individuals, No. (%)		
	With GD and GnRHa (n = 410)	With GD without GnRHa (n = 3820)	General population without GD or GnRHa (n = 73 096)
Assigned sex at birth			
Female	211 (51)	2085 (55)	36 530 (50)
Male	199 (49)	1735 (45)	36 566 (50)
Age at entry date, median (range), y	17.8 (10.7-75.6)	23.1 (8.0-94.2)	22.9 (7.7-94.5)
Individuals with GnRHa treatment	410 (100)	0 (0)	0 (0)
Age <18 y at GnRHa start	219 (53)	NA	NA
Follow-up time after GnRHa start ≥240 d	299 (73)	NA	NA
Individuals with IIH diagnosis	0	2 (<0.1)	8 (<0.1)
Age at first IIH diagnosis, median (range), y	NA	30.6 (23.4-37.9)	25.6 (14.6-68.0)
Follow-up, total, y	940.1	12 762.2	249 422.3
Follow-up, median (range), y	1.4 (0.0-10.1)	2.3 (0.0-11.0)	2.5 (0.0-11.0)
IIH incidence rate per 100 000 person-years, % (95% CI)	0.0 (0.0-392.0)	15.7 (1.9-56.6)	3.2 (1.4-6.3)

Abbreviations: GD, gender dysphoria; GnRHa, gonadotropin-releasing hormone analogue; IIH, idiopathic intracranial hypertension; NA, not applicable.

Among individuals with GD receiving GnRHa, no cases of IIH were identified (Table 2). Among individuals with GD without exposure to GnRHa, 2 individuals developed IIH, with an estimated incidence rate of 15.7 (95% CI, 1.9-56.6) per 100 000 person-years. In individuals without GD and without exposure to GnRHa (n = 73 096), 8 were diagnosed with IIH, with an estimated incidence rate of 3.2 (95% CI, 1.4-6.3) per 100 000 person-years.

## Discussion

In this register-based cohort study, following the entire Swedish population over 10 years, no individual with GD exposed to GnRHa developed IIH. The estimated incidence rate of IIH among adults in Sweden was 0.71 per 100 000 individuals, with a female-to-male ratio of approximately 6:1.^3,4^ The incidence of IIH as an adverse effect of GnRHa treatment is unknown. The FDA warning^1^ is based on postmarketing surveillance data, not allowing for incidence calculations. Our incidence estimates have wide confidence intervals due to the rarity of the outcome.

The FDA warning^1^ applies to leuprolide acetate, nafarelin, histrelin, and triptorelin. Of these, only leuprorelin and triptorelin are prescribed in Sweden. Additionally, buserelin and goserelin, GnRHa medications not included in the FDA warning, are also prescribed in Sweden. Although unlikely, different pharmaceutical formulations could have different risk profiles. According to the FDA report, all 6 individuals developed IIH within 240 days of starting GnRHa and all but 1 had precocious puberty as the underlying condition (the sixth patient had GD). In our study, 73% of individuals with GD and GnRHa treatment were followed up for at least 240 days, thus allowing enough time to capture the outcome of IIH. In our data, no people with precocious puberty developed IIH. Study limitations include the small number of individuals with the outcome and the outpatient register had not achieved full coverage during the study period.

Better-powered studies of GnRHa exposure are needed to elucidate the association between GnRHa and IIH. The data on individuals diagnosed with GD in Sweden between 2006 and 2016 who were prescribed GnRHa do not present substantial evidence to support this association.

## Notes

Supplement 1.
eAppendix.

Supplement 2. Data Sharing Statement
