# Supplementary material for: Incidence of Idiopathic Intracranial Hypertension in Individuals With Gonadotropin-Releasing Hormone Analogue Treatment for Gender Dysphoria in Sweden
Source: JAMA Pediatr. 2023 May 1;177(7):726–7. doi: 10.1001/jamapediatrics.2023.0656 (PMC10152371; doi:10.1001/jamapediatrics.2023.0656)
Supplement: Supplement 1. — eAppendix. [file jamapediatr-e230656-s001.pdf]

Supplemental Online Content

Karamanis G, Frisell T, Holmberg M, et al. Incidence of idiopathic intracranial hypertension in individuals with gonadotropin-releasing hormone analogue treatment for gender dysphoria in Sweden. *JAMA Pediatr.* Published online May 1, 2023. doi:10.1001/jamapediatrics.2023.0656

eAppendix.

This supplemental material has been provided by the authors to give readers additional information about their work.

© 2023 Karamanis G et al. *JAMA Pediatrics.*

eAppendix.

For the purposes of this study, we utilized data that had already been collected from a number of national registers in Sweden. The dataset included all individuals (n=4,480) who had a registered diagnosis of gender dysphoria (GD), as per the 10th version of the International Classification of Diseases (ICD-10), in the National Patient Register (NPR) between 2001 and 2016.

For each individual with GD (the exposed group), ten individuals with the same assigned sex at birth, and ten with the opposite sex at birth, without a GD diagnosis at the time of their index (exposed) individual's GD diagnosis, were randomly selected from the general population, matched for age and residency county. The collection design allowed unexposed individuals to be selected multiple times for different index individuals. There were 83,140 matched individuals (82,327 unique).

For the analysis of this article, we limited the time period to 2006-2016. The National Prescribed Drug Register (PDR) began in July 2005, and therefore, no information on prescriptions was available before this date. By having a six-month interval from July 2005 to January 2006, we minimized the risk of information bias by including only probable new treatment episodes with GnRHa.

After applying the time period restriction, we had 3,821 individuals with GD who were included in the two subcohorts, GD with GnRHa treatment, and GD without GnRHa treatment. Individuals with GD and GnRHa treatment contributed person-time until the dispension of GnRHa treatment to the subcohort of GD without treatment, as described in Table 1.

The remaining matched individuals without GD, after applying the time period restriction, numbered 73,160 (72,413 unique). Of these, 35 (33 unique) were later diagnosed with GD and were excluded from the subcohort of the general population without GD, leaving a total of 73,125 (72,380 unique) individuals.

The first subcohort (n=411) included individuals with at least one GD diagnosis and at least one dispensed prescription of GnRHa after or within two years before the first GD diagnosis. The second inclusion criterion assumes that the indication for treatment with GnRHa was for the management of GD.

The second subcohort (n=3,820) included individuals with at least one GD diagnosis but without a dispensed prescription of GnRHa.

One individual was found to have a first diagnosis of idiopathic intracranial hypertension (IIH) several years before the entry date and was therefore excluded from subcohorts 1 and 2, leaving 410 and 3,820 individuals, respectively, which were the final populations of the two subcohorts.

The third subcohort was defined as the matched individuals without GD. They contributed person-time from the date of the first GD diagnosis of their matched person until the earliest event described in Table 1.
Twenty (19 unique) individuals were excluded because of GnRHa treatment before the entry date of their matched person, leaving 73,105 (72,361 unique) individuals.

Nine (9 unique) individuals were excluded because of an IIH diagnosis before the entry date of their matched person, leaving 73,096 (72,352 unique) individuals, which was the final population of the third subcohort.

© 2023 Karamanis G et al. *JAMA Pediatrics.*

The incidence rates and corresponding 95% CIs were calculated using the PoissonCI() function of the DescTools package in R. The output of the function is given as incidence per 1 person year, which was then multiplied by 100,000 to obtain the incidence rate per 100,000 person-years.

© 2023 Karamanis G et al. *JAMA Pediatrics.*
