# Supplementary material for: Incidence of Idiopathic Intracranial Hypertension in Individuals With Gonadotropin-Releasing Hormone Analogue Treatment for Gender Dysphoria in Sweden
Source: JAMA Pediatr. 2023 May 1;177(7):726–7. doi: 10.1001/jamapediatrics.2023.0656 (PMC10152371; doi:10.1001/jamapediatrics.2023.0656)
Supplement: Supplement 2. — Data Sharing Statement [file jamapediatr-e230656-s002.pdf]

Data Sharing Statement

Karamanis. Incidence of Idiopathic Intracranial Hypertension in Individuals With Gonadotropin-Releasing Hormone Analogue Treatment for Gender Dysphoria in Sweden. *JAMA Pediatr*. Published May 01, 2023. doi:10.1001/jamapediatrics.2023.0656

Data
Data available: No
